# ST-DeepGait: A Spatiotemporal Deep Learning Model for Human Gait Recognition

**DOI:** 10.3390/s22208075

**Published:** 2022-10-21

**Authors:** Latisha Konz, Andrew Hill, Farnoush Banaei-Kashani

**Affiliations:** Department of Computer Science and Engineering, University of Colorado Denver, Denver, CO 80204, USA

**Keywords:** deep learning, gait recognition, spatiotemporal sequence data analysis

## Abstract

Human gait analysis presents an opportunity to study complex spatiotemporal data transpiring as co-movement patterns of multiple moving objects (i.e., human joints). Such patterns are acknowledged as movement signatures specific to an individual, offering the possibility to identify each individual based on unique gait patterns. We present a spatiotemporal deep learning model, dubbed ST-DeepGait, to featurize spatiotemporal co-movement patterns of human joints, and accordingly classify such patterns to enable human gait recognition. To this end, the ST-DeepGait model architecture is designed according to the spatiotemporal human skeletal graph in order to impose learning the salient local spatial dynamics of gait as they occur over time. Moreover, we employ a multi-layer RNN architecture to induce a sequential notion of gait cycles in the model. Our experimental results show that ST-DeepGait can achieve recognition accuracy rates over 90%. Furthermore, we qualitatively evaluate the model with the class embeddings to show interpretable separability of the features in geometric latent space. Finally, to evaluate the generalizability of our proposed model, we perform a zero-shot detection on 10 classes of data completely unseen during training and achieve a recognition accuracy rate of 88% overall. With this paper, we also contribute our gait dataset captured with an RGB-D sensor containing approximately 30 video samples of each subject for 100 subjects totaling 3087 samples. While we use human gait analysis as a motivating application to evaluate ST-DeepGait, we believe that this model can be simply adopted and adapted to study co-movement patterns of multiple moving objects in other applications such as in sports analytics and traffic pattern analysis.

## 1. Introduction

Human gait encompasses a complex set of biomechanical dynamics that are orchestrated by the central nervous system and happen completely at the unconscious level. Research findings from neurophysiology and psychology [1,2] support that gait uniquely captures the discriminative and identifying attributes of the way in which an individual walks. Interestingly, humans can have this experience of seeing a person of familiarity walking at a distance possibly in the opposing direction, and yet have the remarkable ability to recognize who that person is due to the person’s individual gait characteristics. Identifying these gait feature patterns, although easy for humans, still poses a significant research problem for the field of gait analytics. The quantified and interpretated individual gait patterns have significant potential to be used as biometric markers for verification [3], as identification for security or forensic purposes, for analyzing and diagnosing gait anomalies arising from mobility disorders such as neurodegenerative disease [4], or as part of a gait-monitoring system for rehabilitation for stroke patients or fall detection [5,6].

Gait recognition, a method to extract and authenticate signature gait patterns, continues to be a difficult problem. In particular, gait authentication faces the difficulty of measuring time-varying dynamics co-occurring with multivariate spatial relationships that must consider a feature space representation that will embed the measurements of one person with a smaller margin than the measurements of all other subjects. Gait analysis and classification techniques can be, in general, categorized by the modality for capturing the data and its associated representation along with the methods used to process and extract the periodic features to ensure optimal class embeddings. Often, existing solutions place implicit emphasis on a specific domain regarding the spatiotemporal structure of gait parameters used for measurement. For example, RGB video data can be represented as a 2D binary silhouette-based image, often referred to as a Gait Energy Image (GEI) [7,8], where the temporal domain is averaged over all the frames to produce a single image. Although succinct in its representation, GEI maintains the spatial domain, albeit from one viewpoint, but is subject to spatial artifacts such as a coat occluding partial motion of the legs [9]. On the other hand, marker-based and RGB-D solutions look to capture the multivariate positional data of joints over time and often capture the spatial domain by hand-engineering features such as calculating mean anthropometric measurements per person or dynamic gait features such as stride length [10,11].

The framework we have developed to extract features starts by modeling the rich multivariate gait dynamics into a spatiotemporal graph. Since gait can be reduced to a periodic function of symmetry [12] (i.e., right double support (both feet touch the ground, right leg in front), right midstance (legs are positioned closest together, right foot touches the ground), left double support (both feet touch the ground, left leg in front), and left midstance (legs are positioned closest together, left foot touches the ground)), we can encode the co-movement patterns of limb joints directly into the model by giving symmetric moving objects (joints) shared parameter access in the model. After the joint inputs are semantically defined in the graph, a multi-layer Recurrent Neural Network (RNN) is generated to featurize the data. Recurrent Neural Networks (RNNs) [13] are computational graph networks that process sequential data with a recurrent activation at each time-step and have shown powerful capability in many end-to-end tasks. Previous works [14,15] have shown promise in specific use cases such as activity recognition and driver maneuver and human motion forecasting. We propose to use the spatiotemporal RNN architecture to extract the feature embedding space of individual gait signatures for classification. We evaluate our gait recognition framework on two classifiers and consistently achieve over 90% accuracy. Moreover, our extensive experiments demonstrate that by capturing both spatial and temporal physics of the movement patterns in human gait, our physics-based spatiotemporal deep learning model can significantly outperform other existing gait recognition models.

The remainder of the paper is organized as follows. In Section 2, we discuss the related work. Section 3 provides the problem definition and our solution. In Section 4, we describe the model ST-DeepGait in detail. Then, we provide the technical implementation and describe the experimental study, evaluation metrics, and report on the results in Section 5. Finally, we provide our concluding remarks and discuss future directions in Section 6.

## 2. Related Work

Previous research efforts have applied and developed techniques from image processing, data mining, and machine learning to capture the time-varying dynamics of human gait co-occurring in multivariate dimensional space. The difficulty of identifying and recognizing patterns of gait lies in the complexity of data representation and the high dimensionality of the spatiotemporal feature space associated with modeling human motion. In this section, we review the related work according to the data modalities and featurization methods implemented to capture the gait dynamics.

### 2.1. RGB-Based Methods

RGB video-based methods for person identification have been driven by an implicit focus on the spatial domain and are often plagued with problematic feature representations of the 2D imagery due to variations in illumination, point of view, and invariance among same class subjects [9,16]. These traditional approaches are based on extracting handcrafted features from image frames. Most existing work follows a background subtraction method using a binary silhouette as a means for abstracting the walking subject and deriving measurements from its shape. As the subject moves in each frame, the foreground pixels provide movement in subsequent frames as the background pixels are subtracted off.

As noted in Section 1, previously with gait data, Gait Energy Images (GEIs) attempt to integrate the binary silhouette image processing technique with temporal information by averaging the silhouette across all frames of the video [17]. Much work has been conducted in the development and application of GEIs, including metric learning frameworks such as siamese networks [18,19], to help with class invariance with differing viewpoints. Other handcrafted feature methods from 2D look to extract human body parts or skeletal information using distance function approximations as a mean [20]. In this work, the authors show that their skeletal method places greater emphasis on how the body moves, rather than on outside covariate factors as the silhouette-based methods tend to do. As mentioned, silhouette-based methods tend to suffer from artifacts not relevant to body shape and gait patterns such as articles of clothing. For example, a jacket or bag may occlude the actual shape information used for inference especially when sensing from a side viewpoint.

### 2.2. Depth-Based Methods

Many previous works have had the insight to use 3D skeletal data to provide kinematic details out of raw gait data [11,21,22]. The authors in [11] extracted joint-angle trajectories of the lower limbs from the 2D GEI as a way to extend the silhouette-based method to include the kinematic features of joints. Current gait classification research stemming from inexpensive depth sensors such as Microsoft Kinect include [5,10,23,24,25]. Depth sensors, e.g., Microsoft Kinect, provide 3D skeletal tracking technology with high accuracy.

The authors in [10] use statistical methods that involve handcrafting features and random sampling to infer whether the features are relevant to a particular sample. For example, they calculate the mean and standard deviation during a gait cycle for step length as the maximum distance between two ankles, and the stride length as two steps. They also handcraft, for one gait cycle, the mean and standard deviation for the length of bones and the height as the sum of bone lengths from head to foot. Other approaches use Dynamic Time Warping (DTW) as a measure to compare distances between joint signals [25]. For example, in [10], the authors calculate joint pair distances over a gait cycle as a way to interpret relative movements of gait. The authors propose that the relative joint pair distances effectively extract the gait pattern. In addition, the authors calculate the joint relative angles between 3D points by defining a reference point. Their main objective is to encode the relative movement patterns between joints. Then, they perform DTW to align the data series collected from joints. Handcrafted features can inject domain-specific skewness. For example, the practitioners in [10,11] only consider calculating the dynamic features from the angles at the hips, knees, and ankles as they follow a five-link biped model [26]. Without the existence of a standard skeletal data set for classification, the machine learning models are often trained with a small number of samples per class. Unfortunately, this can cause significant bias in the model and is not likely to generalize well to new data sets. Although these depth-based methods move toward considering the dynamic behavior of gait and are human-interpretable, these ad hoc approaches to featurizing data are highly reliant on the training data the model sees, the sample of selected features, and even the value of *k* for nearest neighbors classification, leading to instability during inference. We show in Section 5 that our method for embedding features leads to a generalizable method by testing inference on new classes of data that are completely unseen during training.

### 2.3. Spatiotemporal Deep Learning Methods

Convolutional Neural Networks (CNNs) [27] have been extensively employed for 2D image recognition tasks and are extended toward video recognition tasks such as video-based gait recognition. The authors in [28] argue that many deep learning architectures for video recognition often rely on the early layers to implicitly capture motion dependencies. As mentioned in Section 1, some solutions place inherent emphasis on a particular domain, and as [28] reasons, CNNs tend to place it on the spatial domain. Interestingly, the authors in [29], propose a Convolutional Autoencoder (CAE) to explicitly formulate the temporal domain of the manifold for skeletal human motion modeling by convolving over the time domain. In this CNN-based approach, the joint positions are flattened into a 1D array per each time step, thereby removing the multivariate spatial features of the joint movements. While this model ignores any spatial representation, the authors claim the explicit encoding of the temporal aspect of motion result in equal performance as compared with capturing the pose subspace. In [30], the authors employ IMU sensors placed at five joint positions to capture spatial and temporal patterns of movement. In this work, the authors employ a 2D CNN to model the data represented in the frequency domain (rather original domain); hence, ignoring many important data features. Moreover, this approach requires extensive model calibration and adjustment per subject due to known limitations of IMU sensors. In [14,15,31,32], in various motion prediction and driver maneuver forecasting applications, the spatiotemporal dependencies are considered to model the data for specific tasks. We use spatiotemporal graphs (ST-graph) to encode the spatial relationships that also determine the wiring of the neural network architecture to allow for a rich spatial and temporal representation. We also recognize the periodicity and bilateral symmetry of the human gait data by predefining the co-movement behavior in the ST-graph to guide the training of the neural network based on the physics of the task; hence, introducing a physics-based neural network [33] for human gait recognition.

## 3. Proposed Method

**Figure 1:**
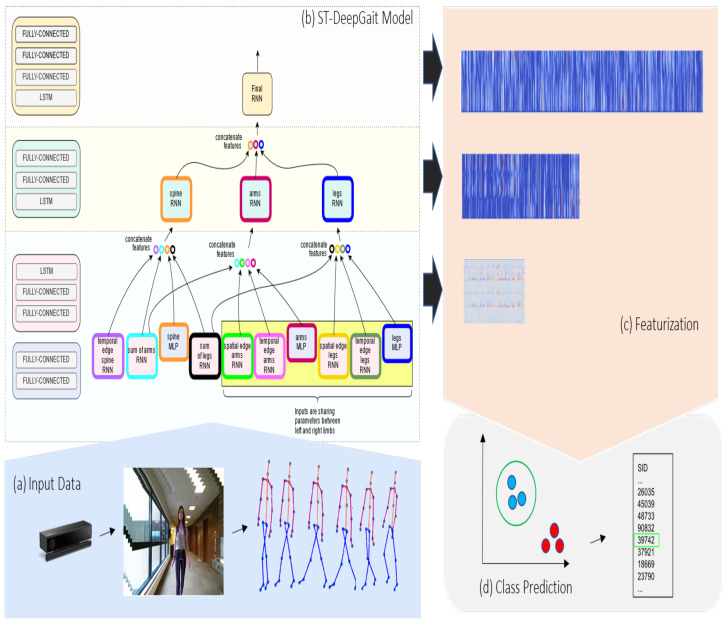
This is a summary of the gait recognition model we developed. (**a**). The raw video is collected from an RGB-D sensor. The skeletal joint rotations are provided by the sensor’s technology, which we preprocess and define in the ST-Graph. (**b**). The deep learning model is constructed according to the arrangement ST-Graph. (**c**). For each layer, the model outputs the feature maps of size (*T*, 128), (*T*, 256), and (*T*, 512). (**d**). At inference, the model makes the class prediction according to the Maximum Likelihood Estimation (MLE) of the model after training.

In this section, we formally define the problem and provide the general details of our gait recognition model as illustrated in Figure 1. Given the various subjects’ raw gait input from the RGB-D sensor, for example, in Figure 1, we aim to identify who the person is by predicting the actual corresponding Subject Identification (SID) label. Thus, our overall objective is to extract, transform, and embed the features from the multivariate spatiotemporal data captured from the depth sensor to perform classification.

### 3.1. Problem Definition

We consider the gait classification problem as a three-part process: (1) data representation, (2) model training for featurization, and finally, (3) inference.

First, we represent the data as a series of multivariate joint rotations. The orientation data captured as a quaternion are translated by an exponential map for which we formally define a unit vector ω in $R^{3}$, which specifies the direction of rotation and θ in R represents the angle of rotation in radians. The quaternion *q* is normalized as $\hat{q}=\frac{q}{\left|q\right|}$ and is a 4D vector $\vec{q}={\left[q_{x}\;q_{y}\;q_{z}\;q_{w}\right]}^{T}$, and specifically, the axis vector ω is defined by $v_{x}=\frac{q_{x}}{\sqrt{1.0-q_{w}^{2}}};\;v_{y}=\frac{q_{y}}{\sqrt{1.0-q_{w}^{2}}};\;v_{z}=\frac{q_{z}}{\sqrt{1.0-q_{w}^{2}}}$ and where $\theta =2cos^{-1}\left(q_{w}\right)$.

For each $X^{\left(i\right)}$, we consider a series of tensors of rank r≥0 and a dimensionality *d*, where d>1 denotes a multivariate series and d=1 a univariate series, all of length *T*. Then, $X^{\left(i\right)}$, is a $R^{T\times r\times d}$ tensor such that $X_{t,k,l}$ is the $k,l^{th}$ component for r>0, otherwise the *l*th component of the *m*th series at time *t*. However, *T* can vary or remain static for all series in $X^{\left(i\right)}$; to reiterate, there is not a constraint on variable length series for input X. In our case, the gait data aree represented in axis-angle rotations in 3 dimensions. The rank of the input tensor will depend on the number of joints related to the predefined ST-Graph. For example, an arm node could contain the shoulder, elbow, wrist, and hand joints. We would consider this a tensor of rank 1 given the 4 features with dimension *d* = 3. Additionally, each time series is of variable length to account for the natural stocahsticity of human movement, so *T* will depend on the number of frames per sample. In this example, we have one arm input defined as a series $R^{T\times 4\times 3}$.

We can consider each target $y^{\left(i\right)}\in Y$ denoting the true label, for i=(1,2,…,m) where *m* is the number of samples. Each $y^{\left(i\right)}$ is the annotation for a corresponding $X^{\left(i\right)}$. To learn the best parameterization that characterizes the multivariate classification given the training set *X* and targets *Y*, an optimization update given by the cost function $J(X^{\left(i\right)},y^{\left(i\right)},\Theta )$, for i=(1,2,…,m) where *m* is the number of samples. $J(X^{\left(i\right)},y^{\left(i\right)},\hat{\Theta }$) must be minimized to select the best approximation of Θ until convergence. Thus, misclassification errors are distributed through backpropagation, which employs taking the derivative of the cost function *J* with respect to the parameters $\hat{\Theta }$. Thus, the classification function $f_{\hat{\Theta }}\left(x\right)$ will predict the correct label $y^{\left(i\right)}$ given an input $X^{\left(i\right)}$, such that:

(1) $$f_{\hat{\Theta }}=R^{T\times r\times d}\mapsto Y.$$

## 4. ST-DeepGait

An important aspect of our approach for identifying signature spatiotemporal patterns of gait is the explicit definitions of the spatial and temporal relationships applied directly within the model. To enforce network understanding of the spatiotemporal dynamics, the two domains are intertwined by defining the spatial adjacency, spatial symmetry, temporal frame differences, and parameter sharing concurrently within the multi-layer network modules and connections between layers. The routing, and thus, updates during optimization, are dependent on the higher-level semantic understanding of spatiotemporal dependencies of gait. Furthermore, the deep learning environment is capable of making optimal featurization decisions without needing to reduce the dimensionality of the data. Interestingly, this will lead to inferences made on the subtleties of gait features in higher dimensional space without the injection of biophysical domain knowledge.

### 4.1. Spatial Representation

After raw data collection and preprocessing, we model the relations between the joint inputs to the network with an ST-graph. As referenced in Section 1, the dynamics of gait involve a symmetric cycle of multivariate movement patterns and our objective is to model the spatiotemporal structure of gait movement explicitly within the network model. This ST structure allows us to integrate RNNs specific to each graph factor, such as a given node or edge, into the multilayer network architecture. The proposed model captures the spatial structure of joint relations through both the hierarchy of joint rotational relativity and through how information is passed and updated according to higher-level semantics throughout the model. We model the co-movement patterns between parallel limbs with the same graph coloring between nodes and edges. For example, we introduce parameter sharing between alike limbs. Parameter sharing in deep learning models provides a method for compressing the model size leading to network efficiency. Additionally, the model updates the parameters via gradients from both limbs further imposing the spatial structure of gait based on the ST-graph definition in Section 4.4.

### 4.2. Temporal Representation

The temporal domain is captured through the hidden layer of the RNN through LSTM (Long Short-Term Memory) cells. LSTMs [34] maintain a context vector with the output vector at each time step. The context vector has a gating mechanism that affects the cell memory, and thus, the output information of the hidden nodes. At each time step, the LSTM can read from, write to, or reset the cell memory. In addition, RNNs allow for variable length sequences as input into the model, so no additional time-scale processing is needed on the raw video data such as zero-padding or linear interpolation.

### 4.3. Inference

After training, the baseline model is able to produce a posterior probability distribution over each class label to infer which label best describes the input given the parameterized $\hat{\Theta }$ and input $x^{\left(j\right)}$, where j=(1,…,N) and *N* is the number of test samples. Inference can be made directly by the same softmax activation Equation (2) outputs; additionally, the model can return the final latent layer, or the embeddings provided by the network, as the learned representation that encodes the geometric embedded features of each class as a continuous vector in lower dimensional space. Our final goal is to construct a feature extractor that can be used to authenticate a test case’s gait. To this end, the model can simply make an inference by the argmax of the posterior probability distribution given by the softmax activation on the logits, or by maintaining the class embeddings to map input $x^{\left(j\right)}$ to a point in *d*-dimensional embedded space such that similar gait inputs are close to one another. From here, a simple (*k*-Nearest Neighbors) *k*NN classification can be conducted to verify that the person is recognizable by test input $x^{\left(j\right)}$.

### 4.4. Formalization

To decompose the spatial and temporal components, we assume an ST-graph $G=\{V,E_{s},E_{t}\}$ to structure the semantic factors for all *m* input series $X^{\left(i\right)}$. An edge $e\in E_{s}$ denotes the spatial relationship between a pair of vertices in *V*, where $e_{s}=\left(u,v\right)$. Then, it follows that an edge $e\in E_{t}$ denotes the temporal relationship occurring at consecutive time steps for some vertex v∈V where $e_{t}=\left(u,u\right)$.

**Figure 2:**
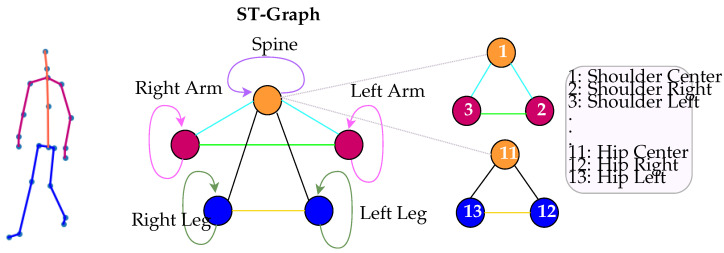
ST-graph example of skeletal joint data. The colors of the skeleton on the left are shown according to the colors of the nodes on the graph. The spine connects to both arms and both legs and this structure will be represented in the design of the model architecture. Each node is designated a temporal edge represented as a self loop, and the arms and legs have spatial adjacency edges to the spine, and spatial symmetry edges to each other. The diagram also shows that the joints are collapsed around a specific node as defined by the human body. For example, *Shoulder Center* and *Hip Center* are both defined to be a part of the spine node. This is specific to our own implementation for computational efficiency. However, the graph is general and can be applied to whatever resolution may be prescribed.

Each node and edge has a corresponding feature tensor with the objective to parameterize them to learn the output $z_{t}$ at each time step in the series. Overall, the parameters are learned to achieve the best approximation of the main objective function in Equation (1). Here, factors can be grouped in such a way that semantically similar vertices can choose to also share factors. This allowance introduces performing parameter sharing over those feature tensors exhibiting equivariance. If we choose to partition vertices on their corresponding semantic meaning, then we can have a distinct set of $A_{V}=\left(V_{1},\ldots ,V_{P}\right)$ where $V_{p}$ denotes a vertex or a set of equivariant vertices and $\Phi_{V_{p}}$ denotes the shared factor function. Then, $A_{E}=\left(E_{1},\ldots ,E_{M}\right)$ make up an edge or the set of edges partitioned such that $E_{m}$ is a set of edges whose pair of vertices belong to some $V_{p}\in A_{V}$. An example is shown in Figure 2.

After partitioning the distinct Φ factors of the ST-graph, we give each Φ factor its own RNN module. $\Phi_{V_{p}}$ for p=(1,…,P) in set $A_{V}$ will be represented as $nodeRNN_{V_{p}}$, and $\Phi_{E_{m}}$ will be represented as $edgeRNN_{E_{m}}$ for m=(1,…,M) in set $A_{E}$. Since $A_{E}$ make up the set of edges incident to pairs of vertices in $A_{V}$, we provide a direct mapping in our computational graph model between the incident edges and vertices with their corresponding edgeRNNs and nodeRNN, respectively. For example, let us consider an edge $E_{m}$ in $A_{E}$ that is incident to $V_{1}$ and $V_{2}$ in $A_{V}$ such that $E_{m}=\left(v_{1},v_{2}\right)$. Thus, the $edgeRNN_{E_{m}}$ feature tensor *Z* will be parameterized and then routed to $V_{1}$ and $V_{2}$.

For spatial representation of V∈G, we create a Multi-Layer Perceptron (MLP) for each $V_{p}\in A_{V}$ such that, given an input $x^{T\times r\times d}\in X$, and parameter $\Theta (W_{r\times d}\times h,b_{h})$, where *W* denotes the weight matrix, and *b* the bias vector, the vertex feature tensors are parameterized by a linear combination of the form $h_{l}\left(x\right)=W_{x}+b$ and $h_{l+1}=h\;\left(h_{l}\right)$ for l=(1,…,L), where *L* is the number of fully-connected hidden layers. Here, an activation function α can be used to introduce non-linearity into the network via linear function *h*, such that $h_{l+1}=\alpha \left(h\;\left(h_{l}\right)\right)$ for l=(1,…,L).

For each edgeRNN, we build an RNN with LSTM cell such that given an input $x^{\left(i\right)}$, a hidden vector $H=(h_{1},\ldots ,h_{T})$ is operated on by a composite LSTM function [34], such that $LSTM(W_{xh}x_{t}+W_{hh}h_{t-1}+b_{h})$ gives the output tensor $Z=(z_{1},\ldots ,z_{T})$, such that $z_{t}=W_{hz}h_{t}+b_{z}$.

For each nodeRNN, we concatenate the spatial output feature map from the MLP activation of $V_{p}$ with each edge factor from edgeRNN feature tensor *Z* that is incident to $V_{p}$. At this point in the network, each nodeRNN contains all relational feature maps from the space and time domains defined by the ST-Graph *G* and output of a single LSTM layer as input. Thus, a second LSTM layer within each nodeRNN receives the feature maps of $e_{s}\in E_{s}$ and $e_{t}\in E_{t}$ that have been defined as jointly interacting with the incident $V_{p}$.

A final nodeRNN concatenates the feature maps from all nodeRNNs represented by the partitioning of $A_{V}$ leading to a third and final LSTM layer. The final nodeRNN receives a softmax activation such that on the output tensor *Z*,

(2) $${\hat{y}}^{\left(i\right)}=softmax{\left(z_{1},\ldots ,z_{C}\right)}_{i}=\frac{e^{z_{c}}}{\sum_{k=1}^{C}e^{z_{k}}}\;\forall \;c\in C,$$

and *C* is the number of classes. The softmax activation squashes the values to non-negative numbers that sum to 1, generating what can be interpreted as a probability distribution over *C* classes. During training, optimization occurs with the Maximum Likelihood Estimation (MLE) of the posterior probability distribution given by softmax minimizing the objective function, which we formulate as the cross-entropy error function such that the distance between the true distribution and the softmax distribution is defined as:

(3) $$L_{CE}=-\sum_{i=1}^{C}\;y_{i}\;\log\hat{y_{i}}.$$

### 4.5. Model Architecture

**Figure 3:**
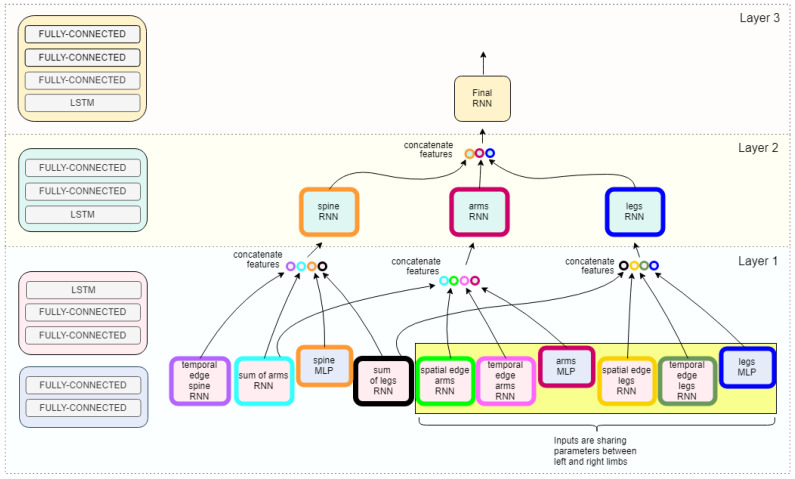
RNN model architecture at time step *t*. The first layer receives the inputs as defined in the spatiotemporal graph in Figure 2. The first layer RNNs characterize the semantic relations between nodes as edges. This includes the spatial structure, as in adjacency, but also in symmetry through parameter sharing in the network. Each color denotes a factor function and the edgeRNNs are colored according to the graph in Figure 2. The parameter sharing is denoted by those edgeRNN modules highlighted within the yellow box. The edges are defined in the network as edgeRNNs in the base layer and are routed to the incident nodes of the ST-Graph. The three colored nodes of the ST-Graph (Figure 2) manifests in the network as three nodeRNNs in the second layer of the model. See Figure 4 for a detailed view of a nodeRNN. Finally, the output from the nodeRNNs are concatenated as input to the last layer’s nodeRNN. Each layer contains an RNN module meaning that at each frame the previous modules context is considered in the next frame allowing for a rich understanding of the spatiotemporal dynamics of gait.

The mapping of the model, as outlined in Section 4.1, is implemented within the framework of the multi-layer RNN architecture. The model network framework is in Figure 3. As shown in Figure 2, the nodes of the graph represent the left and right arm, left and right leg, and the trunk or spine of the body; the edges model pairwise relationships between interacting nodes. For example, in Figure 2, the hip center joint, considered a part of the spine, is connected by a spatial edge to both the hip left and hip right, as defined as the left and right leg, respectively. The model is designed within its inherent structure and routing to learn the relative movements occurring among these joint rotations with the intent to distinguish the variations of these relative movements between classes. For additional model compression and computational efficiency, we collapsed the joints into a single vertex based on correlated movement within the general trajectory of joint rotation occurring in one cycle. This representation reduces the number of RNNs needed at each layer as defined by the nodeRNN and edgeRNN in Section 4.1. Furthermore, as shown in Figure 3, the second layer of the architecture shows three nodes to represent all joint inputs.

**Figure 4:**
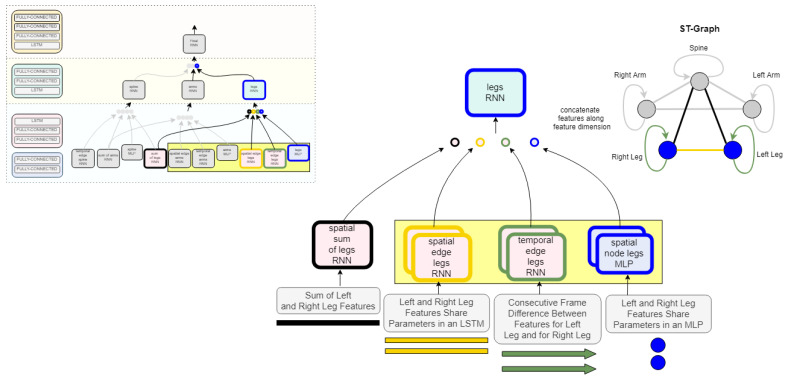
Leg nodeRNN implementation details. This example shows how the input is represented for the leg node of the graph. The edges are defined in edgeRNNs as spatial and temporal information based on the semantics of the ST-Graph in Figure 2. (The model architecture and ST-Graph shown is greyed-out to zoom in on the the leg.) The three edgeRNN modules in the yellow denote parameter sharing occurring in the network between the left and right leg. For example, the spatial edge legs’ RNN will have one set of weights for both the left and right leg inputs. This introduces spatial awareness to the network while also compressing the size of the network.

**Figure 5:**
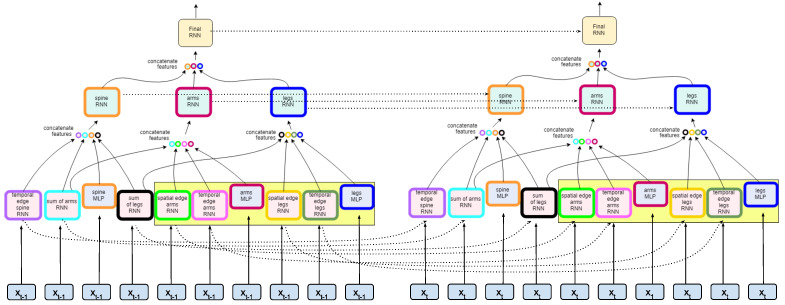
The model unrolled through time. The dotted lines show how the context from the previous LSTMs is passed among the edgeRNNs in the first layer and the nodeRNNs in the second layer and the final nodeRNN in the last layer. At each time step, the spatiotemporal semantics are introduced through the inputs according to the graph but also according to the sequential patterns emerging as it updates via backpropagation through time.

**Figure 6:**
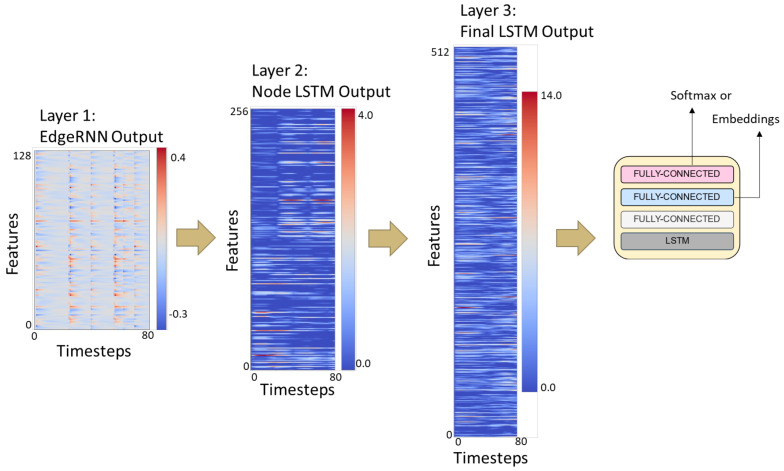
ST-DeepGait model featurization example occurring at each layer. The LSTM feature output at each layer is shown for illustration. The first layer EdgeRNNs have a feature output of the entire sequence length by the number of hidden neurons. The second layer NodeRNNs have a feature output of the entire sequence length by the number of hidden neurons. The final layer is a concatenation of all nodeRNNs for an output of sequence length by the number of hidden neurons. The final LSTM featurization is then used to produce the embeddings or make a softmax distribution. The features are not human-interpretable, but show that the network is learning oscillating feature patterns.

At the base of the model, the MLP and edgeRNNs receive the spatial and temporal inputs as defined by the edges connected to each vertex in $V_{p}$. For example, as shown in Figure 4, the node in the ST-graph defining legs contains the features from three spatial edgeRNN modules and one temporal edgeRNN module as they occur unrolled over time. In Figure 5, the dotted lines show how information flows from each layer unrolled over two time steps. Thus, the leg nodeRNN receives a rich ST representation from the base layer routing through its own temporal edgeRNN, a sum of features edgeRNN, and a spatial edge feature extraction occurring in both an edgeRNN and an MLP network. This process is replicated for all vertices in $A_{V}$ as defined by the ST-graph *G*. Additionally, edgeRNNs can be shared across nodeRNNs; edgeRNN sharing not only adds context to the spatiotemporal relationships occurring in the biomechanics of gait but also makes the network more compact. As the network routes through each nodeRNN, in particular, for the spine, legs, and arms, each nodeRNN in $V_{p}$ routes the transformed sequential feature maps through a second LSTM layer. The outputs *Z* from each edgeRNN are concatenated to form a final sequential feature representation to be processed in a final nodeRNN and a third LSTM layer. In Figure 6, a simplified featurization is shown to illustrate the learned features of the LSTMs at each layer. After the specified recurrent routing ends per series $x^{\left(j\right)}$, the last ouptut $z_{T}$ generated by the final LSTM encodes the logit value, or class score, produced during the feedforward pass defined in Section 3. The logit layer can be used with the softmax activation (see Equation (2)) for optimizing the network during training (see Equation (3)), or for after training during inference. Alternatively, after training, the final layer can also be extracted from the model to produce class embeddings.

## 5. Experimental Study

### 5.1. Dataset

Our method uses our own data set composed of the raw input produced by the RGB-D Microsoft Kinect sensor and Software Development Kit (SDK) from which we collect the skeletal gait data based on 20 joints per frame at 30 Hz. The Kinect sensor has an RGB camera with a 1920 × 1080 pixel resolution and a depth camera based on Time-of-Flight (ToF) sensing via infrared (IR) with a depth resolution of 512 × 424 pixels. To capture data, the sensor does not require careful considerations such as calibration, synchronization of several sensors, or body markers.

The data collection was conducted under the approved Colorado Institutional Review Board (IRB) protocol 18-2563 entirely on the University of Colorado Denver campus. Volunteers were recruited to walk forward, moving in the direction toward the sensor (see Figure 1: Input Data). The sensor can accurately track the skeleton approximately at a distance of 3 meters, which captures about 1.5 to 2 gait cycles. Due to this restriction, we asked each volunteer to repeat this process 30 times; this generates approximately 45 to 60 analog noise-inherent cycles per class. Since the objective of our hypothesis is to classify signature spatiotemporal gait patterns, we collected data from 100 individuals under these same conditions to induce robustness in the evaluation of the model. We did not collect any metadata about the person and anonymized each volunteer’s data by annotating the data with a random SID. All data were reviewed in a 3D visualization as part of the data cleaning.

Each joint contains an (x, y, z)-position in camera coordinate space and an (x, y, z, w)-quaternion. The joint quaternions follow a kinematic tree representation for which we convert each joint by an exponential map as defined in Section 3.1 in the coordinate frame of its parent joint. The resulting joint representation allows for three degrees of freedom at each joint in the form of axis-angle. In biomechanics, the determination of the body’s center of mass is important for defining measurements of movements accurately [12]. Thus, the base reference starts at the hip center joint and is sometimes referred to as spine base; this is aptly defined by the sensor’s technology as this joint is likely to have the least amount, if any, rotation during walking [12]. The end joints (i.e., thumb or foot) do not contain orientation data as they occur at the end of the kinematic chain and are defined by the parent, so these are not included in the inputs. We drop the (x, y, z)-positional data due to being absolute in tracking space and for possible scale variation issues as the person moves toward the sensor. The data are preprocessed by an exponential smoothing filter [35] to reduce the jitter acquired from the sensor while tracking. Formally, the filter is defined as $S_{t}=\alpha \;y_{t-1}+\left(1-\alpha \right)\;S_{t-1}$, where α is the smoothing constant. The constant α was determined by visualizing the 3D reconstructed skeletal data before and after filtering.

Limitations of the data include the tracking range of the sensor. The lack of a standard database for gait data, especially for the case of machine learning methods, might be due to the difficulty of collecting the large amounts of data required to build a high quality machine learning model that will generalize well to test data. For our study, we had each volunteer walk in front of the sensor 30 times in an attempt to accumulate enough data for a deep learning model to generalize well. Additionally, the sensor is subject to error when not in tracking range, or, for example, if one of the joints is not in the field of view. We cleaned the data by ensuring poor estimates of tracked skeletons were not included in the data set.

Our classification problem is about extracting and matching spatiotemporal patterns particular to an individual’s gait. Skeletal parameters such as bone lengths and height certainly could be particular to an individual but are scalar values that could be used to classify by maintaining a lookup table. However, this solution does not capture the spirit of matching dynamic spatiotemporal patterns. We constrain the solution as such to adhere to solving pattern recognition on multivariate spatial data co-occurring with time-varying dynamics. Therefore, our training and inference are made solely on the relative joint rotational data derived from the (x, y, z, w)-quaternion data that make up a gait cycle.

As a research contribution, we release our data set containing around 30 gait videos per 100 subjects totaling 3087 gait video samples. Each frame maintains the raw joint data in both the absolute (x, y, z)-positions and relative (x, y, z, w)-quaternions. In addition, we preprocessed the data with an exponential smoothing filter and converted the quaternions to $R^{3}$ space in the form of axis-angle representation. The data set will both remain in raw and filtered form and in the binary files we used for our own experiments for researchers to reproduce or develop new work with which is available on our lab’s website [36]. All source codes will also be released online with a link to our Git repository upon publication.

### 5.2. Implementation

We implemented ST-DeepGait using the TensorFlow 1.12 Deep Learning (DL) framework. TensorFlow provides efficient automatic differentiation and we did not make modifications to its existing tools. We made use of TensorFlow’s variable_reuse structure to perform parameter sharing between limbs in ST-DeepGait. TensorFlow also allows for saving $\hat{\Theta }$ after training via *model checkpointing*. The model is then restored, with $\hat{\Theta }$ frozen, to produce the class embeddings on the *X* input. This is completed entirely online as a single process. Our system implementation was written in Python 3.6 from the data collection and preprocessing to the evaluation and results. All of our experiments were run on a multi-core cluster containing 16 compute nodes each containing 2 × Intel Xeon E5-2650v4 Broadwell-EP 2.20 GHz Twelve Cores with 128 GB of RAM and 1 GPU node containing 4 Nvidia Tesla P100s. We chose to run the ST-DeepGait model on the compute nodes as it does not contain any convolutional layers and the differences in runtimes were negligible. The bottleneck in runtime mostly stems from the sequential dependency between LSTM layers. The approximate time for training 100 subjects on ST-DeepGait takes about 12–16 h depending on the data split. Inference for the same split takes 5 min.

### 5.3. Model Hyperparameters

The model architecture detailed at each time step is shown in Figure 3. The hidden layers in the base of the model’s edgeRNNs (i.e., LSTM and MLP) each contain 128 neurons. The second layer of nodeRNNs each contains 256 neurons in the hidden layer of the LSTM layer and fully-connected layer. At the final layer, the last LSTM contains 512 neurons. All layers contain a Rectified Linear Unit (ReLU) activation, except that the last layer before the softmax activation has a hyperbolic tangent (tanh) nonlinearity. To obtain class embeddings, we return the hidden layer vector of dimensionality d=128 before the tanh or softmax activation. The network has an Adam optimizer [37] with the learning rate set to 3 ×$10^{-5}$ and updates with Stochastic Gradient Descent (SGD). Since the number of frames never exceed 185 and average around 100, we choose not to truncate unrolling through time. At each epoch, the training data are shuffled so the model is not fed a false pattern within the input queue.

### 5.4. Experimental Methodology

The evaluation of the ST-DeepGait model is two-fold: (1) to consider and evaluate the model quality in terms of stability, generality, and consistency, on the featurization of the spatiotemporal gait data with the class embeddings, and (2) to perform a behavioral analysis of the model.

The objective of the experiments was to learn an optimal $\hat{\Theta }$ such that the correct label predictions could be made on unseen test examples. To this end, the data *X* and corresponding *y* label were split into a training set and test set. We tested the model on two separate splits: (1) 80% training and 20% test, (2) 60% training and 40% test. Each sample contains a series of variable length *T* due to the natural variation between data recordings, and a multivariate feature matrix for the 20 joints each in the axis-angle representation in $R^{3}$. However, the dimension of 20 reduces to the number of joints required to define the input for each edgeRNN such that spine and leg inputs are a $R^{T\times 3\times 3}$ tensor, and arm inputs are a $R^{T\times 4\times 3}$ tensor.

### 5.5. Featurization Evaluation

We evaluated the model on its approximation of geometric properties in latent space and the separability of gait patterns between different classes. The model returns the last hidden layer of dimensionality d=128 from the model after training from which we produce the class embeddings. Thus, the embedding layer learns to organize the gait data in the d=128 embedded space that optimizes the objective function of classification. Specifically, the embedding will map the *X* data such that similar gaits are placed near each other in latent space.

We compared the embeddings of the class features returned from the trained model to a Convolutional-Autoencoder (CAE) [38] and to a handcrafted features method. The CAE was inspired by the approach in Section 2 from the work in [29]. The CAE had the convolutional specifications: Input(100x7x3)-CNN(30x1@25)-tanh-CNN(15x17@20)-tanh-Dense(100)-Softmax. CNNs require a static input size and a scaling for variable length inputs. The input was time-scaled by linear interpolation to a length of 100. Some videos were more than 100 frames and some less; so inputs were downsampled or upsampled, respectively. The CNN also employs a 2 × 1 average pooling and batch normalization. The CAE learns a latent feature vector of dimensionality d=128 on all of the gait data for training. Furthermore, the CAE formulates the series, as in [29], explicitly accounting for the temporal domain by performing a 1D convolution over all joints. However, we tried to add a spatial component by convolving over the first axis *T* in a $R^{T\times r}$ tensor, where T=100 and r=1 for each *x*, *y*, and *z* dimension of the axis-angle representation independently represented as 3 channels. The latent layer was used to output the class embeddings during inference just as the ST-DeepGait model embedding layer does. The handcrafted features method was based on [11] and we performed a 50-fold random subspace sampling over the anthropometric and dynamic gait features, which were calculated from the absolute (x, y, z)-positional data and not the relative rotational data, but the data are considered relative as the distance measurements are static features specific to a SID’s skeleton. For all handcrafted measurements, we also calculated the mean and standard deviation across all frames per measurement for each sample. We removed the samples that were over two standard deviations away from the mean.

**Figure 7:**
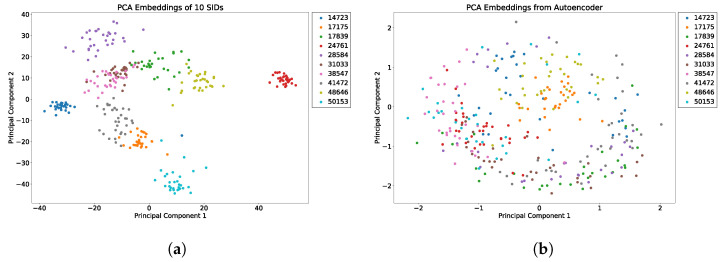
PCA of first 2 principal components for 10 SIDs; 80–20 train–test split. (**a**) ST-DeepGait PCA for the same random 10 SIDs shown by the silhouette score in Table 1 for visualization. (**b**) Convolutional-Autoencoder PCA for the same random 10 SIDs shown by the silhouette score in Table 1 for visualization.

**Figure 8:**
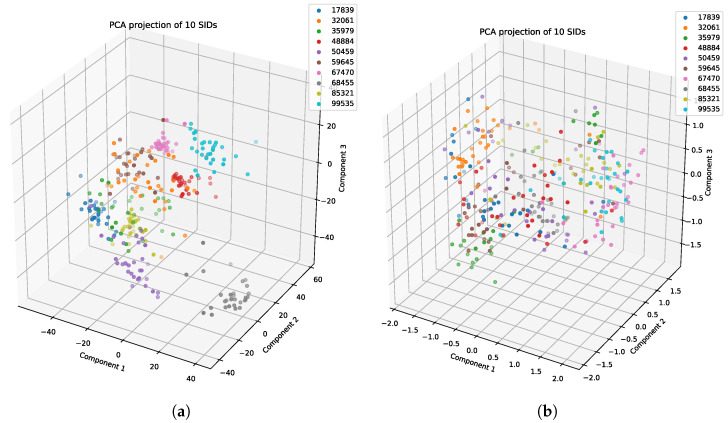
PCA on the first 3 principal components for 10 SIDs 60–40 train–test split. (**a**) ST-DeepGait PCA for a different random 10 SIDs on a 60–40 train-test split for visualization on the first 3 PCs. (**b**) Convolutional-Autoencoder PCA for a different random 10 SIDs on a 60–40 train–test split for visualization on the first 3 PCs.

**Figure 9:**
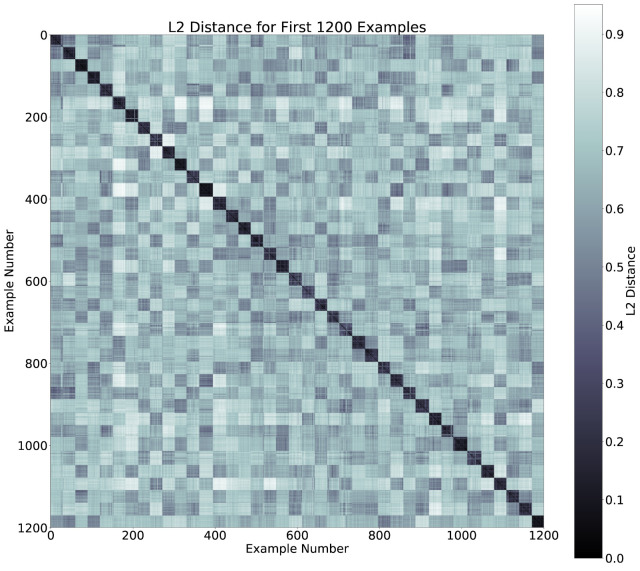
L2 Distance Matrix for first 1200 examples.

**Figure 10:**
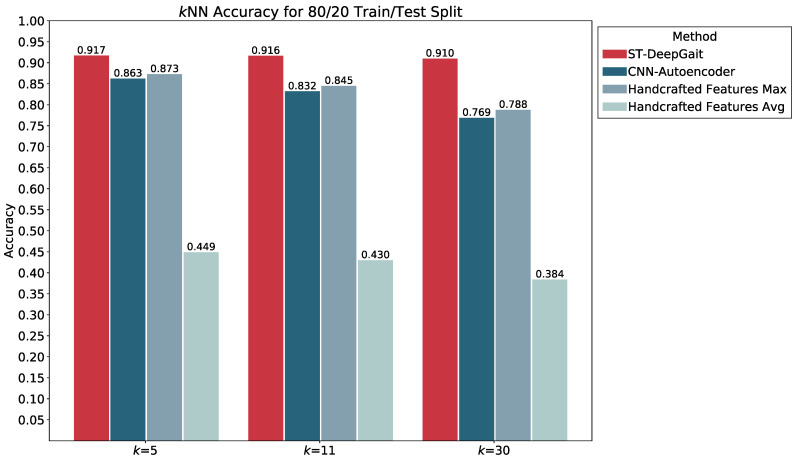
*k*NN classification accuracy for *k* = 5, 11, and 30 on a 80/20 train/test split.

**Figure 11:**
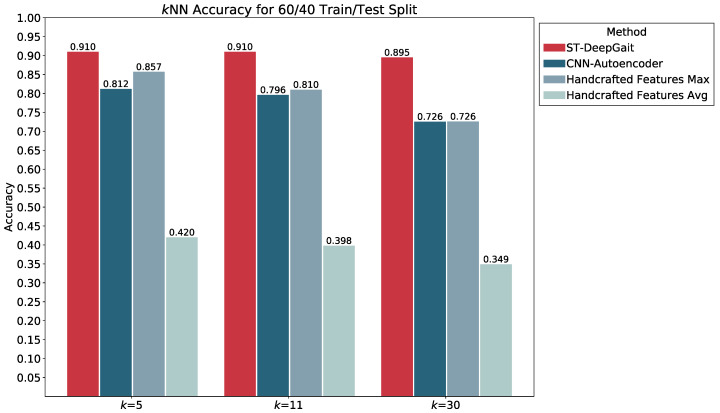
*k*NN classification accuracy for *k* = 5, 11, and 30 on a 60/40 train/test split.

We evaluated the latent space properties with Principal Component Analysis (PCA) in Figure 7a,b and Figure 8a,b. Figure 7a,b represent 10 random SIDs in latent space produced by an 80–20% train–test split. ST-DeepGait showed clear separation and intra-class cluster density on the first two principal components, especially when compared to the Convolutional-Autoencoder. We evaluated the clustering quality with silhouette score [39] shown in Table 1 on the same SIDs in Figure 7a,b. Furthermore, the PCA plots in Figure 7a,b represent a different 10 random SIDs on a 60–40% train–test split. Again, ST-DeepGait showed clear class separation on the first three principal components when compared with the first three principal components of the CAE. We produced an L2 Distance Matrix on all samples and show a subsample of all data to show the granularity of distances between class samples for visualization purposes in Figure 9. The Distance Matrix is ordered by SID and thus shows the approximate 30 samples of each subject with a near-zero distance for intra-class samples. We also include a classifier evaluation by showing the *k*NN classification accuracy for *k* = 5, 11, and 30 of the embedded space. Classification results are shown for the 80–20% train–test split in Figure 10 and for a 60–40% train–test split in Figure 11. The model maintained a stable accuracy even at *k* values of 30 on both train–test splits, whereas the Convolutional-Autoencoder and Handcrafted Features method showed a steady degradation in quality with higher values of *k*.

### 5.6. Behavioral Study

In this section, we provide the comparative results with deep learning models that do not consider both the spatial and temporal domain concurrently. Since the data set is novel, we evaluate the model against a 3-channel CNN and a baseline single-layer LSTM. The specifications for the CNN are identical to the Convolutional-Autoencoder in Section 5.5. The specifications for the LSTM network are: Input(*T*x15)-LSTM(128)-tanh-Dense(100)-tanh-Softmax. Both the CNN and LSTM use the scale-invariant axis-angle joint data except the CNN once again requires a time-scaling via linear interpolation to 100 frames. We chose these models for evaluation to find if a quantitative difference for recognition occurs or if a qualitative boost from the ST-Graph and LSTM occurs when compared with a spatial-invariant CNN without temporal structure or with a sequence-oriented LSTM without spatial structure. We run each model, including ST-DeepGait, for 30 epochs each with a softmax classifier.

**Figure 12:**
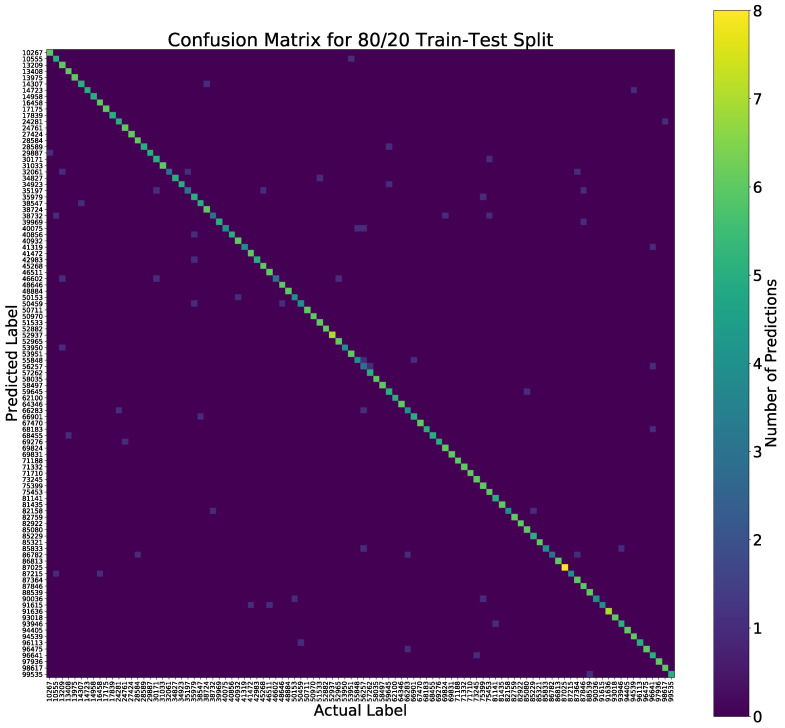
Confusion matrix 80–20 train–test split. The Number of predictions is evaluated on the test set. An 80–20 split yields approximately 6 test examples.

Classification rates were based on the unseen test examples according to the train-test split ratio. Since we collected the data for learning features, the data set has a class balance between all SIDs, and, for example, for a 60% train and 40% test split, the models were trained on approximately 18 examples per SID. During inference testing, this yielded a set of approximately 12 unseen samples per SID. Looking at the base performance in accuracy, the model performed well strictly in terms of classifying ST gait input with the appropriate label, even at 100 class labels. Furthermore, the model appeared to be impervious to false positives and false negatives at the same rate given the metrics in the Confusion Matrix, Precision, Recall, F1, EER, and CMC. The Confusion Matrix carried much of the information for classification and is shown in Figure 12. Furthermore, the training and test accuracy values showed less overfitting than the other models meaning that the model generalizes the patterns learned for the objective of classifying gait patterns relatively well, as shown in Table 2 and Table 3.

**Figure 13:**
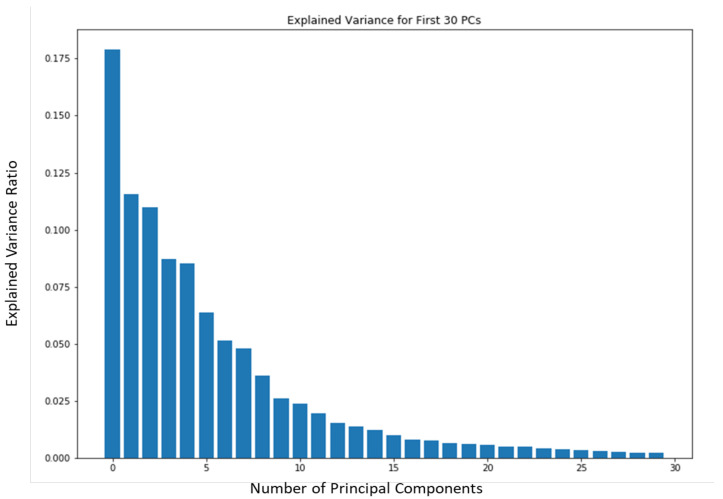
Scree Plot The Scree Plot shows the stability of ST-DeepGait’s featurization in embedded space by showing the explained variance ratio according to the first principal components from the PCA plot in Figure 8a. Interestingly, the ST-DeepGait model appears to have compressed the complex multivariate spatiotemporal joint feature patterns for gait into approximately the first 15 PCs.

To test consistency and stability of the model behavior we evaluated the model feature embeddings on a subspace sampling of the embeddings in latent space. We performed this iteratively to find any brittleness or sensitivity of the selected features in latent space. After randomly selecting a subspace, we performed *k*NN classification to evaluate the stability of the embedded space. After iteratively producing classification F1 scores, the model never really showed degradation until a random subsample of 11 features or less occurred. We produced a Scree Plot of the embedding space on the 60–40% train–test split, as shown in Figure 8a,b. The Scree Plot shows the explained variance ratio based on the eigenvalues according to PCA in Figure 13 and agrees with the subsampling stability.

**Figure 14:**
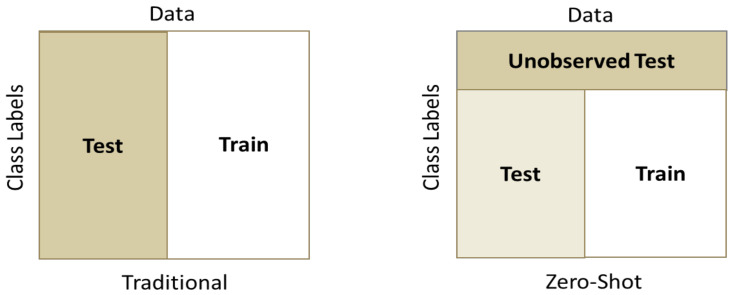
Zero-Shot data split versus traditional data split. For zero-shot detection, the data is split along the class SIDs where a portion are not included or observed during the training of the model. The remaining observed portion still undergoes the traditional train–test split ratio.

**Figure 15:**
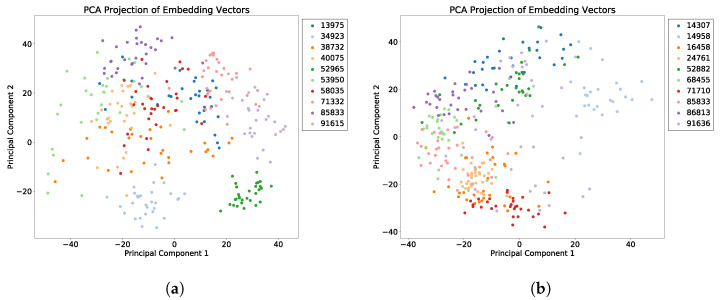
Evaluation of ST-DeepGait model on zero-shot detection on 10% of the class SIDs (not seen by the model during training) in the learned feature space of 90% seen SIDs during training using *k*NN classification. Unseen class SIDs are titrated into the embedded space to check for overall generality of the model. PCA charts show the results for 10 unseen class embeddings in latent space. (**a**) First two principal components on 80–20 train–test split for 10 unseen class SIDs. (**b**) First two principal components on 60–40 train–test split for 10 unseen class SIDs.

**Figure 16:**
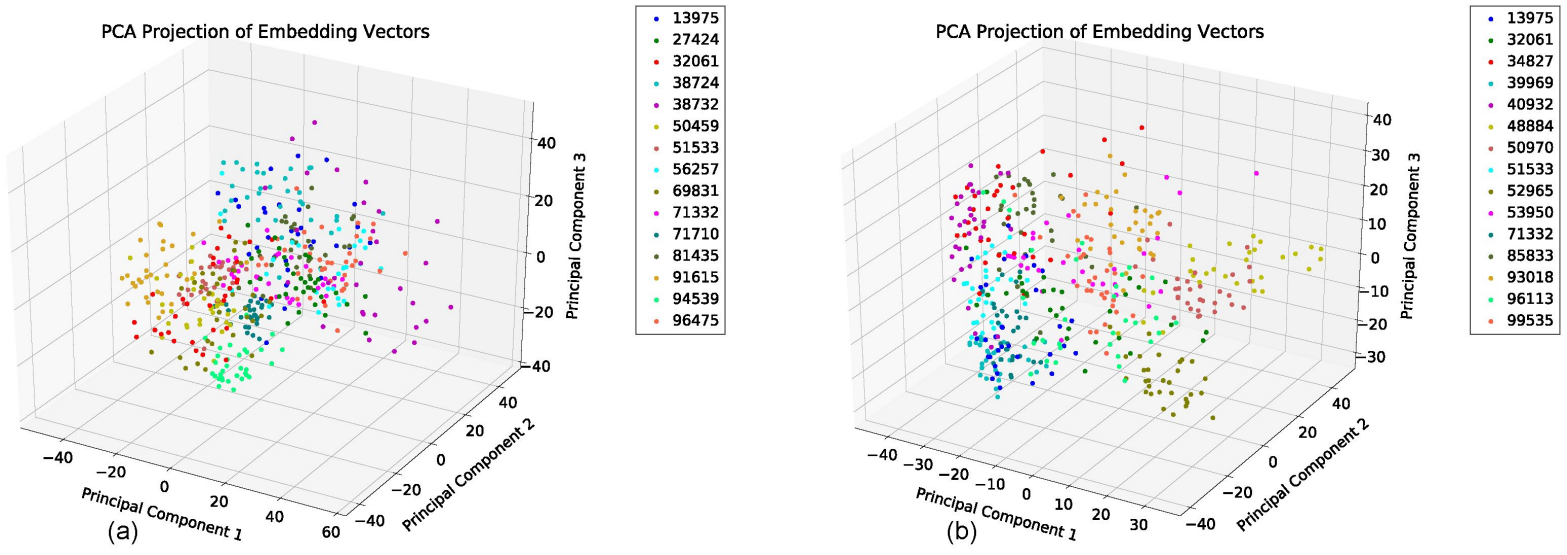
Evaluation of ST-DeepGait model on zero-shot detection on 15% of the class SIDs (not seen by the model during training) in the learned feature space of 85% seen SIDs during training using *k*NN classification. (**a**) First three principal components on 80–20 train–test split for 15 unseen class SIDs. (**b**) First three principal components on 60–40 train–test split for 15 unseen class SIDs.

**Figure 17:**
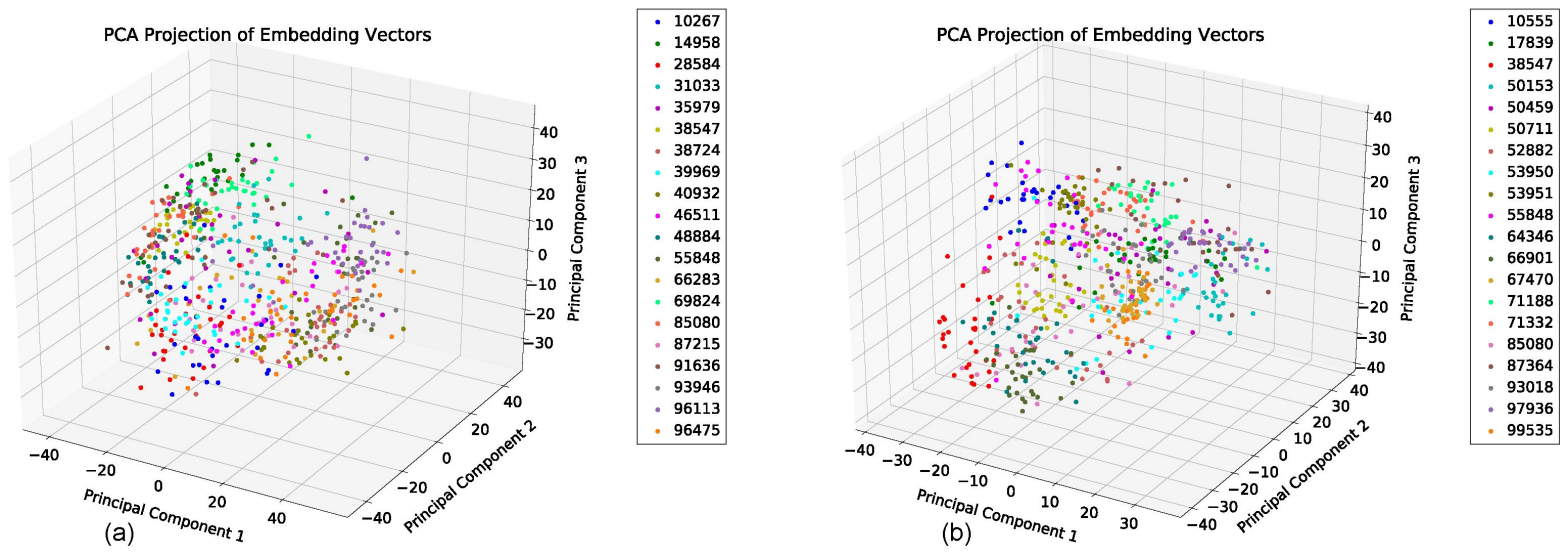
Evaluation of ST-DeepGait model on zero-shot detection on 20% of the class SIDs (not seen by the model during training) in the learned feature space of 85% seen SIDs during training using *k*NN classification. (**a**) First three principal components on 80–20 train–test split for 20 unseen class SIDs. (**b**) First three principal components on 60–40 train–test split for 20 unseen class SIDs.

**Figure 18:**
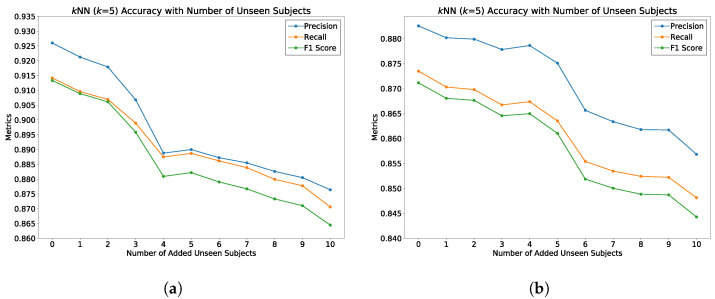
Evaluation of ST-DeepGait model on zero-shot detection on 10% of the class SIDs completely unseen by the model during training in the embedded feature space of 90 seen (during training) SIDs using *k*NN classification. Unseen class SIDs are titrated into the embedded space to check for overall generality of the model. (**a**) The 80–20 train–test split for all data seen and unseen class SIDs; (**b**) 60–40 train–test split for all data seen and unseen class SIDs.

**Figure 19:**
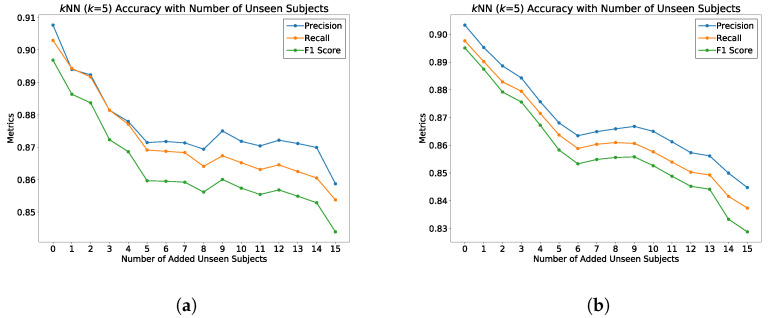
Evaluation of ST-DeepGait model on zero-shot detection on 15% of the class SIDs completely unseen by the model during training in the embedded feature space of 85 seen (during training) SIDs using *k*NN classification. Unseen class SIDs are titrated into the embedded space to check for overall generality of the model. (**a**) The 80–20 train–test split for all data seen and unseen class SIDs; (**b**) 60–40 train–test split for all data seen and unseen class SIDs.

To test the generality of the model on learning an optimal latent space for classifying gait patterns, we evaluated the ST-DeepGait model on completely unseen classes of data during training for which we call zero-shot detection. We wanted to know if the model can embed gait data samples belonging to the same SID with a smaller margin than those of a different SID with left-out, unobserved classes of data during training. To perform this evaluation, we randomly selected 10, 15, and 20 SIDs to leave out of the training of the network on both train–test splits: 80% train-20% test and 60% train-40% test. Figure 14 illustrates how the evaluation was performed on the unobserved test data. Although, as expected, the class separation was not as clear as with training examples, some separation is still convincing in Figure 15a,b. We continually tested the embedded environment on several random left-out SIDs, including 10, 15, and 20 SIDs. The PCA plots are shown for the first three principal components in Figure 15, Figure 16 and Figure 17. The *k*NN accuracy also degraded as expected, but was relatively high considering the model never considered the left-out SIDs’ individual features. Figure 18a,b shows how the titration of unseen classes affects the learned latent space overall, specifically for *k*NN classification. The classification degraded as more unseen classes were added but surprisingly remained in the 84-87% accuracy range for a 90% observed and 10% unobserved class mixture as shown. Additionally, in Figure 19a,b, for an 85% observed and 15% unobserved, the learned latent space remained consistent with the previous random split of 90% observed 10% unobserved while also maintaining a 84–89% accuracy range overall. Consequently, we believe the ST-DeepGait model makes inference convincingly by classifying the general ST feature patterns that it learned were important for the task of gait recognition.

### 5.7. Summary of Results

In summary, the experiments run on the class embeddings show that the model learned an optimal latent space for featurization. The PCA plots in Figure 7a and Figure 8a illustrate a clear separation of the class embeddings. The performance is highlighted when compared to the PCA plot in Figure 7b and Figure 8b produced from the latent space of the CAE. For further measurement, we checked the class embedding silhouette scores in Table 1 and showed a notable difference between the cluster ratios of the ST-DeepGait model and the CAE. We also calculated a distance matrix of the embedded space to illustrate how well the class embeddings separated over 32 classes shown by the distance matrix in Figure 9. Interestingly, a *k*NN classifier was run on the embeddings produced by the model and a marginal increase in accuracy occurred over the baseline softmax posterior probability distribution. The ST-DeepGait model outperformed the CAE and handcrafted features for *k* = 5, 11, and 30. The CAE and handcrafted features also show a steady degradation on *k*NN classification as the model starts considering a majority vote with a higher value of *k*. ST-DeepGait remains at the same accuracy only strengthening its ability to separate class features with stability. To compare the stability of classification with the random subsampling technique that was applied to the handcrafted features, we performed a random subsampling on the 128-dimensional embedding vector and the results repeatedly remained in an 86–94% accuracy range. The accuracy generally did not decrease until a random subsample of an 11-dimensional embedding or less occurred.

## 6. Conclusions and Future Work

Gait recognition remains an interesting and difficult problem for the complex nature of its data. Many approaches have been made in gait analytics tasked with the difficulty of capturing and representing the multivariate spatiotemporal quality such that patterns emerge for various tasks such as classification. Many works have moved away from anthropometric and human-interpretable features and toward modeling the kinematic spatiotemporal features in abstract space with machine learning techniques. We also moved in this direction and demonstrated a robust solution for separating and classifying signature gait patterns with a maximum classification accuracy of 93% and an average classification accuracy of 90% by employing an ST-graph to represent the inputs to a deep learning model. Moreover, we showed the model’s ability to embed the spatiotemporal gait features into geometric latent space in a 128-dimensional feature vector by evaluating the embeddings for class separability. For the task of gait recognition, class embeddings provide a compact representation for storage while naturally maintaining security. If a bad actor were to steal the database of 128 × *n* gait embeddings, where *n* is the number of people, it would be difficult to reverse the model used to embed those features in latent space to gain the identity of each person. The embedded feature space also allows for the ease to apply or compare additional machine learning techniques that we did not cover in this work. Deep metric learning, transfer learning, meta-learning, and other statistical methods could be conducted offline, or more interestingly, integrated into the learning of this model as a way to improve performance or generality. For future work, we would continue testing the environment for performing and generating online machine learning methods for not only improving the accuracy rate, but also for performing in various conditions such as the zero-shot detection test. Generating clusters of the embedded space, or producing other statisitical properties even at each layer, could provide insightful information for the practitioner or the network optimizer to update on, and lead to new ways for learning.

## Figures and Tables

**Table 1:** Silhouette score for class embeddings on a random set of 10 SIDs.

SID	ST-DeepGait	CAE
14723	0.6036	−0.0034
17175	0.5657	0.0876
17839	0.4162	0.0709
24761	0.8215	0.2288
28584	0.4494	−0.0018
31033	0.5893	0.1659
38547	0.3969	0.1579
41472	0.7017	0.0865
48646	0.4435	0.0990
50153	0.3876	0.0737

**Table 2:** Summary of results for classification with Softmax 80–20 train-test split.

Classifier	Train Accuracy	Test Accuracy	Precision	Recall	F1-Score	EER	CMC (k = 5)
ST-DeepGait	98.8%	91.7%	91.5%	91.6%	91.36	0.0034	0.956
LSTM(128)-Dense(100)	99.3%	82.8%	85.5%	83.7%	83.1%	0.0322	0.957
3-Channel CNN	98.6%	76.0%	81.4%	76.0%	76.2%	0.017	0.891

**Table 3:** Summary of results for classification with Softmax 60–40 train–test split.

Classifier	Train Accuracy	Test Accuracy	Precision	Recall	F1-Score	EER	CMC (k = 5)
ST-DeepGait	98.1%	87.8%	88.1%	87.22%	86.9%	0.005	0.96
LSTM(128)-Dense(100)	98.2%	77.4%	79.2%	78.5%	78.2%	0.377	0.94
3-Channel CNN	98.6%	69.1%	72.4%	69.1%	69.1%	0.0386	0.8855

## Data Availability

The dataset used in this study is available at the following site: https://cse.ucdenver.edu/~bdlab/datasets/gait/index.html (accessed on 2 September 2022).
